# The Use of an Assistant

**Published:** 1901-02

**Authors:** A. H. Brockway

**Affiliations:** New York


### THE USE OF AN ASSISTANT.[1]

BY DR. A. H. BROCKWAY, NEW YORK.

We hear much of the advance made in the practice of medicine and surgery within the past few years, and it has, indeed, been great, but especially do we hear of the progress made in that branch which we follow,—dentistry. Answering to the law of development, this itself has come to constantly cover a larger and larger field of usefulness, so that the tendency to divide into specialties, as has the parent profession, is already beginning to be observed. This condition demands on the part of the dentist a larger equipment in the way of instruments and appliances for the proper performance of his work, and their care and best service requires far more of the operator than did the simpler methods of earlier times. This being so, it is proper to inquire if we are

---

[1] Read before The New York Institute of Stomatology, November 9, 1900.

neglecting any means whereby we may increase our efficiency in this direction. I have long thought that many of us are doing so, and I wish briefly to point out in what respect. I refer to the employment of an assistant at the chair.

Of none of the improved appliances which have come into use —the engine, the rubber dam, the separator, the matrix—can the best service be got by one working unaided and alone. Take, for instance, the burring-engine: see how great an advantage it must be to have some one to manage it, leaving the operator to stand firmly on both feet, free to give his whole attention to the work he is doing, instead of balancing unsteadily on one foot, as most do, their bodies swaying with the movement of the propelling leg. Or, take the adjustment of the rubber dam, I have many times seen the dentist working alone spend from five to fifteen minutes doing this when with a trained assistant it could have been done, and with far less annoyance to the patient, in a tenth of the time consumed.

But many will assert that they are quite able and satisfied to do these things unaided. Able they may be, but satisfied they ought not to be.

Some years ago a country surgeon in a small backwoods settlement, in an emergency, alone and unaided, by the light of only a candle, successfully performed the Cæsarian operation. This, of course, showed great skill and courage; but how much lessened would have been the chance of failure could he have had the aid of modern hospital methods,—electric light and three or more helpers to wait on him. And yet there are many dentists, without this poor surgeon's excuse of necessity, going on in much the same inefficient way. Their attitude reminds me of a self-satisfied and unprogressive physician of whom I heard not long since, who, having a serious case of typhoid fever under treatment, scouted scornfully the suggestion that he have the assistance of a trained nurse, declaring that for his part he had rather see the devil enter a house than a trained nurse. It is gratifying to learn that the anxious relatives promptly took the case out of his hands and called in a man of more progressive ideas.

I am asked what kind of an assistant I prefer, and what are the specific duties required of one? My own experience has been considerable, having constantly employed one or more for the past twenty-five years. I have had them of both sexes, but, all things considered, I prefer a young lady of at least sixteen or eighteen years. Such a one, if bright and intelligent, can soon be taught to become exceedingly helpful, and, barring the ever-present danger of lapsing into matrimony, is likely to remain an increasing satisfaction to the dentist and not less so to his patients, who soon come to regard her services as almost indispensable.

Now, how does my assistant assist? In the first place, she stands at the chair as constantly as I do myself, on the opposite side. When the patient has been seated she deftly arranges a large napkin about the neck to prevent soiling of the dress, then lightly smears the lips, especially the corners of the mouth, with refined vaseline to prevent chafing,—a very grateful precaution; she gently holds the lips out of the way while I make examination or during the preliminary cleansing of the teeth, always the first step in putting the mouth in order.

When the preparation of cavities is begun, she starts and controls the engine, and, if the cavity is to be cut out wet, as is often desirable, she throws on the bur from a syringe a stream of tepid water, keeping it from heating and enabling it to cut much cleaner and with greater ease, placing in the mouth during the process the saliva-ejector to relieve annoyance.

Should it be desired to cut out the cavities dry, she assists in adjusting the rubber dam or the cotton rolls; she helps to wipe out the cavity, using her own tweezers, and placing therein a proper obtundent; she prepares ready to my hand most filling-materials, and assists in their introduction; in short, in a hundred ways she serves to lighten my labor and conduce to the comfort of the patient.

In addition to all this she looks after my instruments and appliances, sees that the engine and hand-pieces are kept in order, and when any instrument has been used, that it is cleansed and returned to the proper place ready for its next service.

I have thus briefly indicated some of the duties of an assistant at the chair, and I must say that I consider the employment of one of the greatest advantage. The marvel to me is that so few of our number avail themselves of such a means of lightening their labor and so increasing their efficiency.

Some doubtless are kept from it by their dislike to having a third person about the chair, many by the natural *vis inertia* which clogs most of us and restrains from entering untried paths, but I trust that what I have said may not be wholly unprofitable to those who have honored me with their presence and attention, and that it may prove more persuasive than was the sermon which St. Anthony preached to the assembled and delighted fishes, who all

"Swam up harmonious
To hear Saint Antonious;
No sermon beside,
Had then so edified."

But

"The sermon now ended,
Each turned and descended;
The pikes went on stealing,
The eels went on eeling;
Much delighted were they,
But preferred the old way!"

